# Unzipping the Secrets of Amyloid Disassembly by the Human Disaggregase

**DOI:** 10.3390/cells10102745

**Published:** 2021-10-14

**Authors:** Aitor Franco, Lorea Velasco-Carneros, Naiara Alvarez, Natalia Orozco, Fernando Moro, Adelina Prado, Arturo Muga

**Affiliations:** 1 Department of Biochemistry and Molecular Biology, Faculty of Science and Technology, University of the Basque Country UPV/EHU, 48080 Bilbao, Spain; aitor.francob@ehu.eus (A.F.); lorea.velasco@ehu.eus (L.V.-C.); naiara.alvarez@ehu.eus (N.A.); nataliaorozko@hotmail.com (N.O.); fernando.moro@ehu.eus (F.M.); adelina.prado@ehu.eus (A.P.); 2 Instituto Biofisika (UPV/EHU, CSIC), University of the Basque Country, 48940 Leioa, Spain; 3 Fundación Biofísica Bizkaia/Biofisika Bizkaia Fundazioa (FBB), Barrio Sarriena s/n, 48940 Leioa, Spain

**Keywords:** neurodegeneration, amyloid, chaperone, disaggregase

## Abstract

Neurodegenerative diseases (NDs) are increasingly positioned as leading causes of global deaths. The accelerated aging of the population and its strong relationship with neurodegeneration forecast these pathologies as a huge global health problem in the upcoming years. In this scenario, there is an urgent need for understanding the basic molecular mechanisms associated with such diseases. A major molecular hallmark of most NDs is the accumulation of insoluble and toxic protein aggregates, known as amyloids, in extracellular or intracellular deposits. Here, we review the current knowledge on how molecular chaperones, and more specifically a ternary protein complex referred to as the human disaggregase, deals with amyloids. This machinery, composed of the constitutive Hsp70 (Hsc70), the class B J-protein DnaJB1 and the nucleotide exchange factor Apg2 (Hsp110), disassembles amyloids of α-synuclein implicated in Parkinson’s disease as well as of other disease-associated proteins such as tau and huntingtin. We highlight recent studies that have led to the dissection of the mechanism used by this chaperone system to perform its disaggregase activity. We also discuss whether this chaperone-mediated disassembly mechanism could be used to solubilize other amyloidogenic substrates. Finally, we evaluate the implications of the chaperone system in amyloid clearance and associated toxicity, which could be critical for the development of new therapies.

## 1. Introduction: Neurodegeneration and Amyloid Deposition

**Figure 1:**
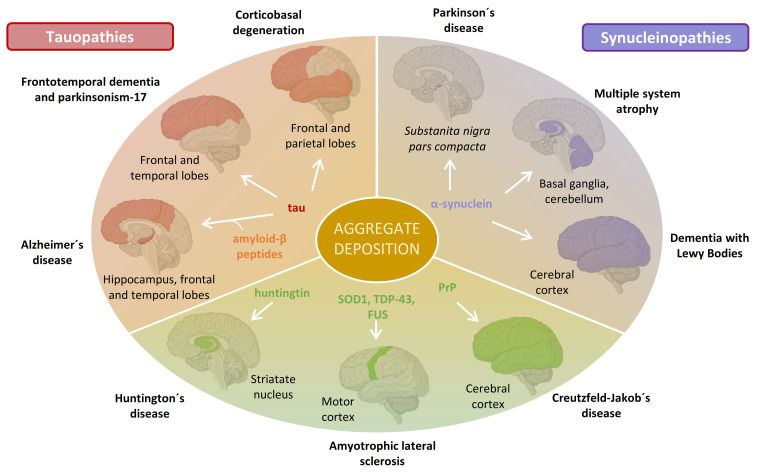
Protein aggregate deposition disorders affecting the nervous system. A multiplicity of proteins has been found forming aggregate deposits in the brain of patients with NDs. Depending on the disease, the amyloidogenic protein that forms such deposits and their location in the brain vary. Aggregates of the same protein can be found in different brain regions resulting in different disorders, as found for tau and α-syn. NDs involving the aggregation of tau or α-syn are referred to as tauopathies and synucleopathies, respectively. This figure illustrates the main brain locations of amyloid deposits of some amyloidogenic proteins, which may fluctuate with the progression of the disease or among patients.

Neurodegenerative diseases (NDs) have a multifactorial etiology resulting from an intricate relationship between genetic and environmental factors [1]. A histopathological hallmark of most NDs include the deposition of aggregated proteins called amyloids into extracellular or intracellular inclusions [2]. The identity of the conformationally altered protein and the anatomical brain regions and cell types affected differ depending on the disease (Figure 1). For instance, Alzheimer´s disease (AD) is characterized by the deposition of two distinct types of aggregates in the temporal and parietal lobes of the brain: extracellular plaques composed of amyloid-β (Aβ) peptides (cleavage products of the transmembrane protein APP), and intracellular neurofibrillary tangles composed of tau proteins [3]. In Parkinson´s disease (PD), aggregates of the protein α-synuclein (α-syn) accumulate inside dopaminergic neurons of the *substantia nigra pars compacta* [4]. Deposits of superoxide dismutase 1 (SOD1) [5], TAR DNA binding protein (TDP-43) [6], RNA-binding protein FUS [7], huntingtin (Htt) [8], or PrP prion protein [9] have been identified in different NDs (Figure 1).

Protein misfolding and its deposition into proteinaceous aggregate inclusions have a two-faced pathomechanism: a loss of physiological function and a gain of toxic function [10]. Misfolding and aggregation of proteins imply the loss of their native structure, and therefore, their physiological function. This may include loss of catalytic activity or loss of interactions with partner proteins or ligands [11,12,13]. Additionally, the generation and accumulation of misfolded protein species also involves a gain of toxic function as these aggregation-prone species have a heightened tendency to engage in inappropriate interactions with other cellular components [2]. Through both mechanisms, aggregates alter fundamental cellular processes, ultimately leading to cell death.

## 2. Synucleinopathies and α-Synuclein Amyloid Formation

The synuclein family consists of three members: α-, β-, and γ-synuclein [14,15]. Since their discovery, the *SNCA* gene, which encodes for α-syn, has been the focus of intensive research due to its association with neurodegeneration. α-syn is very abundant in neurons, being primarily located at the presynaptic termini [16]. Although it represents 1% of the cytosolic proteins, α-syn can also be found bound to synaptic membranes [17]. This protein is believed to play a major role in the regulation of synaptic trafficking, homeostasis, and neurotransmitter release by interacting with both synaptic vesicles and synaptic proteins such as phospholipase D2, members of the RAB small GTPases family, and SNARE complexes [18]. Other cellular processes in which α-syn is involved include signal transduction, mitochondria functioning, and oxidative stress regulation [18].

Aggregation of α-syn into amyloid fibrils and their subsequent accumulation into intracellular inclusions is a hallmark of many neurodegenerative disorders collectively known as synucleinopathies (Figure 1). Within this group, dementia with Lewy bodies (DLB), multiple system atrophy (MSA), and PD are the most common [19]. In PD and DLB, inclusions are preferentially found inside neurons and are called Lewy bodies (LBs) and Lewy neurites (LNs) [20], while in MSA, these inclusions are localized in glial cells as poorly organized bundles of fibrils and are referred to as glial cytoplasmatic inclusions [21]. Although the general belief was that α-syn was the major component of these intracellular inclusions, recent studies have revealed that their composition is highly heterogeneous, containing cytoskeletal elements, membrane fragments, vesicles, lysosomes, mitochondria, and other misshaped organelles [22,23]. Aggregation of α-syn plays a key role in their formation by constituting a scaffold of fibrillated protein that sequesters all these cellular components [24]. Although it is currently accepted that the toxicity of amyloids mainly comes from the oligomeric species (see Section 5), formation of LB-like inclusions has also been suggested to be a driver of neurodegeneration by disrupting cellular functions such as organellar trafficking and inducing mitochondrial damage, all contributing to synaptic dysfunctions [22,24].

The amyloid aggregation pathway of α-syn begins when the protein misfolds and starts clustering. The species formed at this initial stage are composed of a small number of molecules that normally retain the structure of the native protein, being either highly disordered [25] or α-helix-enriched [26,27] intermediates. Only relatively weak intermolecular interactions are involved within these early aggregates and thus, they are typically unstable. However, such aggregates can undergo an internal reorganization to form more stable species with a β-sheet secondary structure, a conversion step often accompanied by an increase in compactness and size in a global process called nucleation [25,28]. These β-structured aggregation nuclei (also known as seeds) can further grow by self-association or through monomer addition (elongation or polymerization) [25,28,29] forming fibrils with a highly regular in-register cross-β structure. Besides primary nucleation, elongation, and self-association, other secondary processes are significant in amyloid assembly [30]. Of particular interest are secondary nucleation reactions including fibril surface-catalyzed formation of new clusters of monomers [31] and fibril fragmentation into smaller pieces [32]. Both secondary processes seem crucial in the formation of additional aggregation intermediates [33], which are the main cytotoxic species of the amyloid formation process [34].

## 3. Disaggregation of α-Synuclein Amyloids by Molecular Chaperones

Molecular chaperones are key effectors of the protein homeostasis network, guiding proteins along productive folding pathways, avoiding and reversing misfolding/aggregation, and cooperating with the degradation machinery [35]. There are several evolutionary conserved families of molecular chaperones that are classified by their molecular weight including the ATP-independent small heat shock (sHsps) and Hsp40 proteins and several ATP-dependent chaperone families (Hsp60, Hsp70, Hsp90, Hsp100) [36,37]. Such a variety allows the modulation of native/aggregate protein states through several mechanisms including de novo folding, prevention of aggregation, neutralization of aggregates, disaggregation, or protein degradation [38].

Chaperone machines known as disaggregases extract proteins from aggregates that can either be targeted to degradation or reactivated, sparing the energetic burden of novel biosynthesis. Bacteria, protists, plants, and fungi possess a powerful bi-chaperone disaggregase composed of the ring-shaped AAA+ chaperone Hsp100 (ClpB in bacteria, Hsp104 in yeast, and Hsp101 in plants) and the Hsp70 system [39]. Hsp100 cooperates with the Hsp70 system to thread trapped polypeptides in an ATP-dependent manner through its central pore [40,41,42], thereby resolving protein aggregates. Metazoan lacks Hsp100 orthologs, and thus the disaggregation activity is chiefly provided by the Hsp70 system [43].

### 3.1. Hsp104 Disaggregase

Hsp104, an AAA+ ATPase from yeast, is composed of six protomers that form an offset hexameric barrel [41]. This disaggregase remodels diverse amorphous (non-amyloid) and amyloid clients by substrate binding and translocation through its central pore [44]. Despite not being a natural substrate of Hsp104, α-syn amyloids are targeted by this disaggregase [44,45]. Hsp104 efficiently disassembled fibrils composed of WT α-syn or the pathogenic point-mutants A30P and A53T [44,45]. However, it disaggregated the variant S129E, a mimetic mutation of α-syn phosphorylated at S129, with lower efficiency [45], and was unable to remodel the E46K pathogenic mutant [44,45]. This chaperone was also shown to disaggregate preamyloid oligomers of α-syn A30P [45], although inactivation of two Hsp104 subunits ablated the activity. In the case of A30P fibrils, inactivation of one subunit was enough to hamper disassembly [44], suggesting that the highly ordered structure of amyloids requires a global cooperative mechanism of ATP hydrolysis and substrate binding at the six subunits to generate the force necessary to extract monomers [44]. This is a completely different mechanism than that used to resolve amorphous aggregates, in which Hsp104 hydrolyzes ATP in a probabilistic mode upon aggregate binding, being a single active subunit within the hexamer enough to drive protein disaggregation [44]. The versatility and powerful activity of Hsp104 has led us to propose its use as a therapeutic disaggregase of α-syn and other amyloids [46].

### 3.2. The Human Hsp70-Based Disaggregase System

Despite the lack of an Hsp100 homologue, the mammalian cytosol possesses a potent, ATP-dependent protein disaggregase and reactivation activity [43]. This activity was shown to be provided by the central chaperone Hsp70 in collaboration with a specific subset of J-domain proteins (JDPs or Hsp40s) and nucleotide-exchange factors (NEFs), a ternary system capable of solubilizing a wide range of amorphous aggregates [43,47,48,49]. Regarding amyloids, equimolar concentrations of the constitutive Hsp70 (HSPA8/Hsc70), the canonical class B J-domain protein DnaJB1 (Hdj1) and the NEF of the Hsp110 family Apg2 were shown to disassemble α-syn fibrils in a timescale of weeks [50], an activity that was further potentiated by the addition of Hsp104 [43,50] or/and the sHsp αB-crystallin [50]. A later study revealed that a chaperone complex composed solely of members of the Hsp70, Hsp40, and Hsp110 families (henceforth called human disaggregase) was able to efficiently reverse α-syn amyloid fibrils within hours [51]. The amount of the NEF was proposed to be the main reason to explain the time-scale differences as substoichiometric levels of the NEF relative to Hsc70 seem critical for good performance [47,51].

Protein disaggregation by the human disaggregase is initiated by JDPs, recognizing and binding to protein aggregate surfaces [52]. JDPs then recruit Hsp70 to the aggregate through the simultaneous interaction with both the substrate and Hsp70, which results in the stimulation of ATP hydrolysis at the Hsp70 nucleotide-binding domain (NBD) [53]. ATP hydrolysis is coupled to a conformational cycle defined by a large-scale reorganization of the Hsp70 substrate-binding domain (SBD), in which the alpha-helical lid subdomain closes over the β-sandwich substrate binding pocket in the ADP state, resulting in substrate capture [54,55]. Although in all JDPs the interaction of the J-domain is responsible for the activation of Hsc70, the adjacent glycine-phenylalanine rich domain (GF) of DnaJB1 blocks the Hsc70-binding site [56]. The interaction of a second site (at the CTDI) of DnaJB1 with the Hsc70 C-terminal tail releases the GF-domain and therefore its inhibitory effect [56]. This auto-inhibitory regulation found in class B JDPs is not present in members of other JDP classes and constitutes an important mechanism in the disaggregation of amyloids, favoring a high density recruitment of Hsc70 to the aggregate surface [56]. In the absence of DnaJB1, Hsc70 decorates the fibrils randomly [51]. However, the multivalent interaction of DnaJB1 with the fibrils allows for the organized recruitment of Hsc70 in a crowded state [57].

Crowding of Hsc70 molecules at the surface of α-syn fibrils has an initial entropic energy barrier that is overcome by the DnaJB1-stimulated ATPase activity of Hsc70, which slows down the dissociation of the Hsc70(ADP) state and thus, increases an order of magnitude the affinity of the chaperone for fibrils [51,57]. Such binding reduces the conformational space accessible due to an excluded volume effect caused by the physical barrier formed by the aggregate [57]. In an attempt to restore conformational freedom, and the associated increase in entropic energy, Hsc70 applies a pulling force away from the aggregate surface that drives aggregate dissolution [57]. This entropic pulling mechanism has also been used to explain Hsp70-mediated clathrin uncoating and protein translocation [58,59]. After ATP-hydrolysis, the timely release of the trapped polypeptides from the fibrils is mediated by a NEF, which stimulates ADP exchange by ATP [60]. Rebinding of ATP and simultaneous opening of the substrate-binding pocket dissociates substrates from Hsp70, promoting localized polypeptide unfolding–refolding events and resetting Hsp70 for the next cycle of substrate binding. Among the several existing Hsp70 NEFs, Bag1, Hsp105a, and Apg2 have been shown to stimulate Hsc70-mediated disassembly of α-syn fibrils, although with very different efficiencies (Bag1 < Hsp105a < Apg2) [51]. The higher molecular mass of NEFs from the Hsp110 family (Hsp105a and Apg2) seems to be the reason for these differences, as disassembly of amyloids is favored by a bulky NEF like Apg2, which besides exchanging ADP by ATP, also rearranges the fibril-bound Hsc70 molecules, increasing crowding and the formation of productive disaggregase assemblies [57].

### 3.3. Models for the Disassembly of Amyloids by the Human Disaggregase

**Figure 2:**
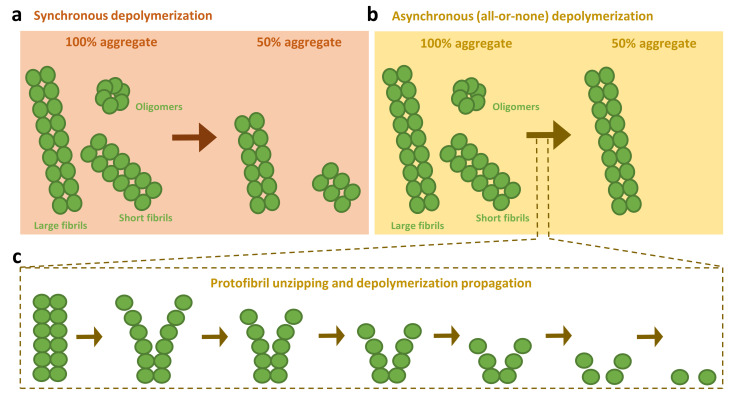
Disassembly models proposed for the human disaggregase. Two depolymerization models have been proposed for the amyloid disassembly activity of the human disaggregase machinery. The first one established that disaggregation was expected to synchronously decrease the length at a similar rate in all fibrils, regardless of their size (**a**). A change in paradigm has recently been put forward, considering the disassembly as an asynchronous process (**b**). In this model, solubilization is not interpreted as a population of aggregates with decreasing size over time, instead being considered as a population with a decreasing number of aggregates over time. This mechanism especially applies for toxic oligomers/short fibrils, being eliminated by the chaperones one by one rather than progressively reducing their size. Disassembly starts with the destabilization of the fibril tips and rapidly progresses to completion through protofilament unzipping and depolymerization, without accumulation of new harmful oligomeric intermediates (**c**). This mechanism is hampered for large fibrils due to their increased stability and reduced number of ends, biasing the human disaggregase activity toward small aggregate species, avoiding their toxicity and growth into less tractable species.

From a mechanistic point of view, there is uncertainty about how human chaperones proceed in the disassembly of amyloids. By using α-syn fibrils capped with his-tagged protomers, Shorter and colleagues showed that Hsc70, Hdj1, and Apg2, in the presence of HspB5, liberated only his-α-syn into the soluble fraction, which indicated that disassembly proceeded via depolymerization [50]. A later contribution by Bukau´s group showed a fast disappearance of longer fibrils with a concomitant appearance of shorter species in the presence of Hsc70, DnaJB1, and Apg2, and proposed that apart from depolymerizing, the human disaggregase could also extract monomers from the center of the fibrils, therefore breaking them into smaller fragments [51]. This was proposed under the premise that a depolymerization-only disassembly might be expected to decrease length at a similar rate in all fibrils. We have recently shown that the disassembly process is the result of an all-or-none abrupt solubilization of individual aggregates (Figure 2), which starts with the destabilization of the fibril tips and rapidly progresses to completion through protofilament unzipping and depolymerization [61]. The fast propagation of the solubilization process is consistent with entropic pulling forces provided by Hsc70 crowding at the fibril surface. Through this mechanism, the Hsc70-based system preferentially disaggregates shorter and thus, more toxic, fibrils of α-syn into monomers, without the accumulation of new harmful oligomeric intermediates. We also showed that this system efficiently eliminates α-syn oligomers kinetically trapped during the self-assembly process, which are similar to the transient oligomeric forms generated at early aggregation stages [25,62], and are especially toxic due to their membrane binding and disruption activities [62,63]. In the case of long fibrils, disaggregation was severely impaired, possibly due to the interruption of the fast depolymerization process [61], which would render monomers and shorter fibrils without altering the number of fibril ends.

Although we do not discard that the human disaggregase could eventually fragment long fibrils of α-syn [51], we reason that its activity relies predominantly on a fast asynchronous depolymerization, targeting small and toxic oligomer aggregate species (Figure 2). This is further evidenced by the fact that the large size of Apg2 limits its ability to act on Hsp70 molecules that are densely packed onto the fibrils [57]. Thus, due to the excluded volume effects, the NEF activity of Apg2 could be biased to less crowded fibril regions such as the ends. At this position, besides stimulating the exchange of ADP by ATP on Hsc70, Apg2 could reposition the fibril-bound Hsc70 molecules in closer proximity to each other, thus increasing the formation of productive disaggregase assemblies [57] and drive protofibril unzipping and depolymerization [61]. A preprint work also points toward this scenario, suggesting that Hsc70 clusters at the fibril ends where monomers are extracted, avoiding intermediate fragmentation steps [64].

## 4. Expanding the Human Disaggregase Activity

### 4.1. Chaperone-Mediated Disassembly of Other Amyloidogenic Proteins

Even though most of the studies related to amyloid disassembly by the human disaggregase published to date have focused on α-syn amyloids, this system can also revert fibrils of other amyloidogenic proteins linked to neurodegeneration [65,66,67]. Huntington’s disease (HD) is a ND caused by an expanded CAG trinucleotide repeat at the N-terminus of the *HTT* gene that results in amyloid formation. The human disaggregase was shown not only to completely suppress fibrilization of HttExon1Q48, but also to solubilize Htt fibrils in vitro [67]. More importantly, all three chaperones of the system (Hsc70, DnaJB1, and Apg2) interacted in vivo with Htt aggregates and depletion of any of them led to enhanced aggregation. Among the components of this chaperone complex, the J-protein was shown to be the concentration-limiting factor and its overexpression in HEK293T was sufficient to strongly ameliorate HttExon1Q97 aggregation [67].

Tau is a microtubule-associated protein encoded by the *MAPT* gene located at chromosome 17. Upon alternative mRNA splicing of this gene, six tau isoforms of different lengths (352–441 amino acids) are produced [68]. Under pathological circumstances, tau proteins fibrillate and form intracellular deposits that can be found in several NDs collectively known as tauopathies (Figure 1). The human disaggregase has been shown to disassemble in vitro produced fibrils from all six tau isoforms [66] and the K18 construct containing the four microtubule-binding repeats [65]. As with α-syn and Htt, the active chaperone complex was formed by Hsc70 and Apg2 in the presence of DnaJB1, although DnaJB4, another member of the class B JDPs, was equally capable of promoting disassembly of tau 1N3R fibrils [66]. Class A JDPs DnaJA1 and DnaJA2 could not assist tau disaggregation [66], reinforcing the specificity of class B JDPs in amyloid disassembly [56]. Chaperone-mediated disaggregation of tau fibrils occurred without fibril fragmentation and generated mainly monomeric protein, although dimeric and tetrameric tau species were also found [66]. This is in good agreement with our data, as the disaggregation efficiency of tau 2N4R dramatically increased upon sonication [61], suggesting a depolymerization process that starts at the fibril ends.

### 4.2. Impact of Amyloid Polymorphism in Chaperone Activity

Despite the differences in the sequence of α-syn, Htt, and tau, the human disaggregase can disassemble fibrils of the three proteins, suggesting a universal and specific targeting mechanism toward the amyloid fold. Amyloid fibrils are characterized by an extended β-sheet secondary structure in which individual β-strands are arranged in an orientation perpendicular to the fiber axis, a structure known as cross-β. It is increasingly evident that the same polypeptide sequence can adopt several pathological conformations, known as amyloid polymorphs [69]. Polymorphs may differ in the number of protofilaments and their organization (protofilament interface and orientation, twist-degree between protofilaments) [70], the number of strands and the identity of the residues involved in the cross-β structure, the orientation of the interacting β-sheets (parallel versus antiparallel intermolecular alignment), the nature of the interactions between β-sheet layers, and the conformation of non-β strand protein regions [71].

**Figure 3:**
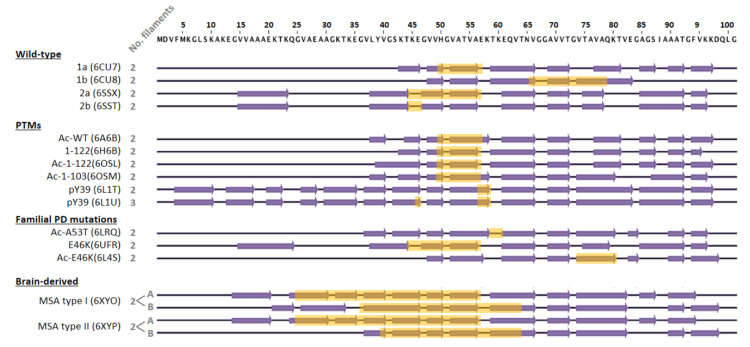
Schematic representation of amino acids 1–100 of α-syn, comparing the secondary structure elements that form the protofilament cores within the different fibril structures solved by cryo-EM. The structures of wild-type, post-translationally modified species, and familial PD mutants were obtained from recombinantly expressed α-syn. In these cases, fibrils were composed of two identical protofilaments or three in the case of fibrils phosphorylated at tyrosine 39 (pY39). The interface between protofilaments is highlighted in yellow. For the WT protein sequence, four polymorphs have been found with three different β-sheet arrangements. Polymorphs 2a and 2b have the same β-sheet arrangement, but differ in the protofilament interface. Regarding PTMs, both N-terminal acetylation and C-terminal truncation seem to have little effect on the secondary structure of the fibril, with a comparable β-sheet arrangement to polymorph 1a. Similarly, neither H50Q nor A53T mutations significantly disturb the 1a fold, only changing the pairing geometry of the protofilaments. In the case of the E46K mutation, two structures have been published, one similar to the 2a polymorph and another one, acetylated at the N-terminus, with a different and more stable fold. Structures of two distinct fibrils (type I and II) derived from brains of deceased MSA patients differ from those obtained in vitro. In these cases, fibrils are composed of two different protofilaments (A and B) that interact with each other through different residues and show much more extended interfaces. β-strand elements were considered as in Schweighauser et al. (2020).

Different structures have been reported for WT α-syn fibrils, revealing that aggregation might generate several polymorphs [72]. Depending on the aggregation conditions, several polymorphs have been produced in vitro, namely 1a, 1b, 2a, or 2b [73]. In most atomic structures known to date, fibrils with a diameter of ~10 nm are composed of two identical protofilaments. The main differences between polymorphs are the number of β-strands, the residues involved in their formation (Figure 3), and the amino acids that participate in the interface between protofilaments (from 2 to 12 residues) [74,75]. Interestingly, residues E46, H50, G51, and A53 are located at this interface in specific polymorphs (Figure 3, interfaces highlighted in yellow), stressing the relevance of point-mutations related to neurodegeneration in fibril formation and growth. In fact, the structures of fibrils carrying the E46K, H50Q, and A53T disease-associated mutations have revealed that they change the protofibril interface or even promote a different fibril fold [75,76,77,78,79].

Post-translational modifications (PTMs) that show different profiles among NDs could also be implicated in the final structure of the fibril. This has been proven for tau, for which cross-talk between PTMs influences filament structure, contributing to the structural diversity of tauopathy strains [80]. Among the PTMs associated with α-syn, acetylation, phosphorylation, and truncation are predominant [81,82,83]. Two fibril structures have been proposed for in vitro produced acetylated α-syn fibrils with an arrangement closely resembling that of polymorph 1a [84,85] and 2a [75]. Most of α-syn is phosphorylated at S129 in the brain of patients that suffered from synucleinopathies [86]. Recombinantly produced pS129 fibrils had a 2a-like structure [75]. pY39 α-syn fibrils showed a completely different fold with a core composed of residues 1 to 100 and fibrils with either two or three protofilaments [87]. Regarding α-syn truncation, C-terminal truncated variants presented a similar fold to the 1a polymorph (Figure 3), but they had an increased helical twist, generating a more packed core [84].

A recent study showed that the structure of α-syn fibrils obtained from MSA patients differs from those assembled from recombinant proteins [88]. MSA-derived fibrils are formed by the asymmetrical packing of two pairs of different protofilaments, with a protofilament interface spanning more than 25 residues from each non-identical protofilament. In addition, a different conformer of assembled α-syn exists in DLB, although its atomic structure has not been solved yet. Similarly, amyloid fibrils derived from post-mortem diseased-brains have identified differently folded forms of tau [89,90,91,92]. Altogether, these results have led to the hypothesis that each ND may be characterized by its own unique amyloid fibril structural signature [88,93].

The effect of amyloid polymorphism in chaperone-mediated disaggregation remains poorly understood. Considering the unzipping-depolymerization disassembly mechanism employed by the human disaggregase [61], factors such as the number of protofilaments, their asymmetric interaction, the interface between them and other specific structural features could affect chaperone binding and/or disaggregation activity. Tau filaments from Braak stage VI AD cortex grey matter prepared using sarkosyl extraction were efficiently solubilized by the human disaggregase [66]. However, the activity of this system to disassemble recombinantly produced C-terminally truncated α-syn fibrils was abolished due to a deficient binding of DnaJB1 [57], indicating that some PTMs could indeed alter the activity of chaperones. Future investigations should focus on the activity of the human diseaggregase against disease-associated polymorphs, which could help us to better understand the molecular basis of their contribution to pathogenesis.

## 5. Human Disaggregase in Amyloid Toxicity and Propagation: Friend and Foe

The toxic function of amyloids primarily arises from the intermediate oligomeric species that populate the aggregation process [34]. Their higher hydrophobicity and smaller size, which results in a high diffusion coefficient, allow them to establish aberrant interactions with different cellular components and spread more readily [2]. It was initially proposed that amyloid deposits could be a protective mechanism to neutralize oligomeric species and reduce their associated toxicity [94]. Nonetheless, amyloid deposits are far from harmless and contribute to toxicity through indirect mechanisms. For instance, it has been shown that key components of the protein homeostasis network that attempt to clear such deposits can be sequestered, reducing the cell power to cope with aggregation [95,96,97,98,99,100,101]. Additionally, mature fibrils serve as a reservoir of aggregate intermediates. Although the amyloid structure is energetically highly favorable [102], amyloid fibrils are not static entities, but exist in equilibrium with monomeric and oligomeric species [103]. Spontaneous fragmentation and secondary nucleation are well-established mechanisms by which mature fibrils can generate new intermediate species [32,104,105,106,107]. Indeed, such processes seem to play a key role in the spreading of the disease within an organism, as these small-sized aggregates can be transmitted between cells and within organs.

**Figure 4:**
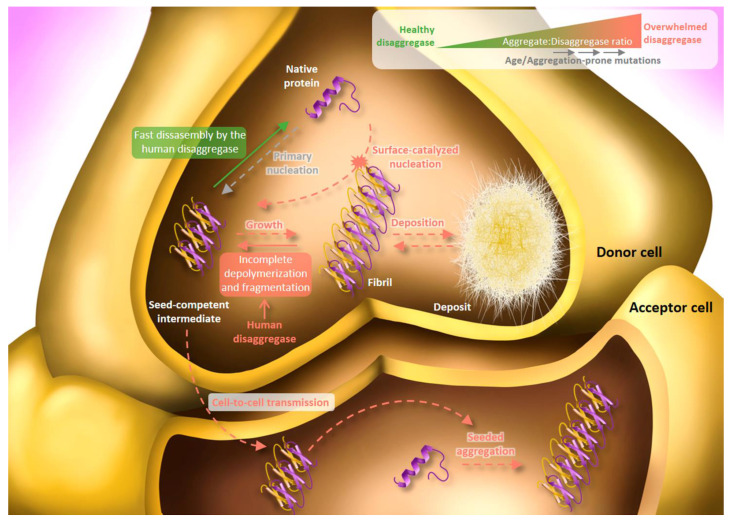
The two faces of the Hsc70-based human disaggregase. In a healthy cell state (low aggregate:disaggregase ratio; green arrow), the human disaggregase rapidly disassemble seed-competent intermediates into the native state, avoiding their toxic effect. Under aggregation stress (high aggregate:disaggregase ratio; red arrows), the disaggregase machinery becomes overwhelmed and the chaperone-mediated disassembly of seed-competent intermediates slows down or is even halted, increasing their life-span. Thus, these species are stable enough to express their toxicity via aberrant interactions with different cellular components and spreading to neighboring cells where seeded-aggregation is induced. Alternatively, oligomers can grow into large fibrils that either tend to deposit or contribute to secondary aggregation processes such as surface-catalyzed nucleation or fibril fragmentation, functioning as a pool of new seed-competent oligomers. The overwhelmed human disaggregase could also contribute to the production of new toxic intermediates in an attempt to disassemble fibrils due to their incomplete depolymerization/fragmentation. Solid arrows represent processes in which the Hsc70-based machinery is actively implicated, while dashed ones occur regardless of its disaggregase activity.

Recent studies propose that the human disaggregase may also be implicated in the prion-like propagation of amyloidogenic proteins [66,108]. Specifically, Tittelmeier et al. showed that depletion of Hsp110 in *C. elegans* impaired Hsp70 disaggregation activity, prevented re-solubilization of amorphous aggregates and compromised the overall cellular folding capacity [108]. At the same time, knockdown of the NEF in young individuals expressing either α-syn or 35 polyglutamine (Q35) fused to Yellow Fluorescence Protein (YFP) reduced foci formation and toxicity, and in the case of α-syn, it also diminished cell-to-cell transmission. These results led the authors to propose that the Hsp70-based disaggregase could promote spreading via amyloid fragmentation similarly to Hsp104, whose fragmentation activity [109,110] is essential for the maintenance of prions in yeast, producing smaller seeds that are more efficiently transmitted to daughter cells [111,112]. Based on our model [61], another possible interpretation would be an incomplete chaperone-mediated disassembly. Although we have shown that the human disaggregase targets the most toxic amyloid species and disassembles them through an all-or-none depolymerization process [61], we have also observed that depolymerization of longer fibrils is not as efficient and may be interrupted half-way through, thus producing shorter, and potentially more toxic, species [61]. Results obtained with tau also point to this interpretation, as fragmentation was not observed for fibrils of this substrate and they were not fully disassembled, generating soluble products (containing monomers, but also dimers and tetramers) that seeded aggregation in P301S tau-expressing HEK293 cells [66]. Thus, the human disaggregase may function as a first barrier against newly acquired oligomeric species, avoiding their toxicity and growth into less tractable species, but overwhelming conditions for the system could favor the aggregation cascade (Figure 4). In this context, an insufficient amount of disaggregase relative to aggregated species might also be a determinant factor to consider. Our group estimated that disaggregation rates of toxic oligomers and short fibrils are rather low at α-syn:Hsc70 molar ratios higher than 2 [61], thus increasing the half-time life of these toxic species. Induced expression of α-syn or Q35 in *C. elegans* [108] might tip the scale toward the aggregate, resulting in high aggregate to chaperone ratios and thus, slower/more inefficient disassembly by the disaggregase.

On the other hand, overexpression of Hsp110 has also been shown to be beneficial in mice models [113,114]. Although the human disaggregase is an important factor in amyloid clearance, in vivo, this process may be more complex due to the activity of other branches of the proteostasis network, which may be interconnected with the Hsp70 system. For instance, αB-crystallin might be implicated in seed spreading, although it is difficult to assess its final effect, as has been shown to both promote [115] and inhibit [116] fibril fragmentation. Kandasamy and Andrésson showed that yeast Hsp110 interacts with the proteasome via its regulatory 19S particle and that NEF inhibition causes accumulation of Hsp70-bound proteasome substrates [117]. The authors suggested a mechanism in which Hsp110 first binds the 19S particle and then recruits the substrate-Hsp70 complex to the proteasome, where Hsp110 promotes nucleotide exchange in Hsp70, mediating substrate release and degradation. This finding is especially relevant considering that N-terminal Ub modification on tau and α-syn enables proteasomes to target and remove their oligomeric assemblies [118]. Furthermore, the proteasome holoenzyme possesses an ATP-dependent fibril-fragmenting activity that reduces the size of large tau and α-syn fibrils into smaller entities [119]. Despite the key function of the proteasome in the protein quality control network, under severe aggregation protein stress, it could play an opposite role by fragmenting and producing more seeds in cells [120]. Autophagy has also been shown to switch roles between seeding and aggregate clearance depending on the level of autophagic flux [121,122].

Aging is undoubtedly the major risk factor for aggregate-related diseases. Molecular chaperones are involved in numerous cellular processes and the increasing load of misfolded/aggregated proteins might become overwhelming (Figure 4). This was evidenced as the available concentration of the human disaggregase was shown to be limiting for the suppression of fibril formation of Htt in the presence of other substrates [67]. Furthermore, the rescue of α-syn or Q35 toxicity by the knockdown of Hsp110 observed in younger animals was lost in older ones, suggesting that the increasing burden of endogenous misfolded proteins that comes with age masks the chaperone-mediated promotion of α-syn and Q35 toxicity [108]. Apart from the increasing amount of aggregates, accumulating evidence suggests a progressive deterioration of the proteostasis network with aging [123] including a downregulation of chaperone expression [124] and the decline in both the proteasome activity and autophagy [125,126]. The age-related increase of oxidative stress and a decrease in mitochondrial function and ATP production could further reduce the efficiency of the disaggregase system. Another important risk factor in NDs are aggregate-prone mutations in amyloidogenic proteins [2], which are normally related to an early onset of the disease. In these cases, the available chaperone pool would be at its full capacity, without the age-related burden of cellular misfolding. However, the great amount of amyloids relative to chaperones would favor an incomplete/slow disassembly and thus, the disaggregase could help spreading, explaining the severity of such cases. This scenario is nicely reproduced in young individuals of *C. elegans* expressing α-syn or Q35 as explained above [108]. Due to these factors, the proteostasis network becomes progressively overwhelmed, leading to aberrant interactions and aggregate deposition. Aggregate deposition is also a cause of the proteostasis decline, seeding further aggregation and sequestering components of the proteostasis network, which interferes with their standard housekeeping functions and additionally favors aggregation. As a result, the proteostasis network drives into a vicious cycle that ultimately leads to its collapse [35,99].

## 6. Conclusions and Future Perspectives

We are progressively deciphering the molecular mechanisms behind the process of amyloid clearance. The human disaggregase is one of the key systems of the proteostasis network that participate in this task. We advocate for a protective action of this machinery against small amyloids, reverting their toxic effect and blocking their conversion into larger aggregates. However, an overwhelmed disaggregase system could have the opposite effect, leading to long-lived, partial disassembly products that could feedback aggregation within the same cell or spread to neighboring ones. In the cellular context, toxicity and prion-like propagation of amyloids could be the combination of multiple intricate sources including not only chaperones, but also the protein degradation systems (proteasome and autophagy). Future investigations should account for the relationship of the different components of the proteostasis network in handling amyloidogenic substrates. In addition, given the multiple fibril polymorphic structures reported to date and the association of some of them with a specific neuropathology, forthcoming research should focus on the impact of amyloid polymorphism in the activity of the different modulators of aggregation/disaggregation. Along these lines, we will gain a better understanding of the molecular determinants of amyloid formation/clearance and toxicity/propagation, which could in turn help us identify new therapeutic targets.

The possibility of enhancing the activity of the human disaggregase as a therapeutic approach is not straightforward, as it must be balanced and ensure that aggregate solubilization is a fast reaction free of potentially toxic intermediates. An activated disaggregase would promote this situation but, as reported for Hsp104, a strong activity could also unfold non-pathogenic proteins, and therefore be toxic, especially if the concentration of the target substrate protein(s) decreases [127]. It would also be desirable to enhance the activity of the specific chaperone-cochaperones combinations involved in the disaggregation of a specific substrate protein, a task that is far for being achieved for the human disaggregase and has started to be developed for Hsp104 [46,128]. This could be accomplished by engineering new chaperones/cochaperones that recognize certain substrates and/or by small molecules that favor specific interactions between the components of the disaggregase and activate the complex. These strategies could be used alongside conventional inhibitors of amyloid aggregation. For instance, trodusquemine, a naturally-occurring aminosterol, can not only inhibit primary and secondary aggregation processes [129], but also effectively suppresses the toxicity of α-syn and Aβ oligomers [129,130]. Similarly, several α-helical peptidic scaffolds have been found to abrogate oligomer-induced cell damage without interfering with the native function of α-syn [131]. These compounds could avoid the deleterious impact of the disaggregases, neutralizing the toxic, seed-competent species released during the disassembly process. Such combinatorial therapeutic strategies could suppose a new tool against increasingly prevalent NDs.

## References

[1] Sheikh S., Haque E., Mir S.S. (2013). Neurodegenerative diseases: Multifactorial conformational diseases and their therapeutic interventions. J. Neurodegener. Dis..

[2] Chiti F., Dobson C.M. (2017). Protein misfolding, amyloid formation, and human disease: A summary of progress over the last decade. Annu. Rev. Biochem..

[3] Villemagne V.L., Doré V., Burnham S.C., Masters C.L., Rowe C.C. (2018). Imaging tau and amyloid-β proteinopathies in Alzheimer disease and other conditions. Nat. Rev. Neurol..

[4] Ozansoy M., Başak A.N. (2013). The central theme of Parkinson’s disease: α-synuclein. Mol. Neurobiol..

[5] Pansarasa O., Bordoni M., Diamanti L., Sproviero D., Gagliardi S., Cereda C. (2018). SOD1 in amyotrophic lateral sclerosis: “Ambivalent” behavior connected to the disease. Int. J. Mol. Sci..

[6] Hergesheimer R.C., Chami A.A., de Assis D.R., Vourc’h P., Andres C.R., Corcia P., Lanznaster D., Blasco H. (2019). The debated toxic role of aggregated TDP-43 in amyotrophic lateral sclerosis: A resolution in sight?. Brain.

[7] Aksoy Y.A., Deng W., Stoddart J., Chung R., Guillemin G., Cole N.J., Neely G.G., Hesselson D. (2020). “STRESSED OUT”: The role of FUS and TDP-43 in amyotrophic lateral sclerosis. Int. J. Biochem. Cell. Biol..

[8] Caterino M., Squillaro T., Montesarchio D., Giordano A., Giancola C., Melone M. (2018). Huntingtin protein: A new option for fixing the Huntington’s disease countdown clock. Neuropharmacology.

[9] Gill A.C., Castle A.R. (2018). The cellular and pathologic prion protein. Handb. Clin. Neurol..

[10] Winklhofer K.F., Haass C. (2008). The two faces of protein misfolding: Gain- and loss-of-function in neurodegenerative diseases. EMBO J..

[11] Ghosh S., Salot S., Sengupta S., Navalkar A., Ghosh D., Jacob R., Das S., Kumar R., Jha N.N., Sahay S. (2017). p53 Amyloid formation leading to its loss of function: Implications in cancer pathogenesis. Cell Death Differ..

[12] Yang H., Li J.-J., Liu S., Zhao J., Jiang Y.-J., Song A.-X., Hu H.-Y. (2014). Aggregation of polyglutamine-expanded Ataxin-3 sequesters its specific interacting partners into inclusions: Implication in a loss-of-function pathology. Sci. Rep..

[13] Budini M., Romano V., Quadri Z., Buratti E., Baralle F.E. (2015). TDP-43 Loss of cellular function through aggregation requires additional structural determinants beyond its C-terminal Q/N Prion-like domain. Hum. Mol. Genet..

[14] Surguchov A. (2015). Intracellular dynamics of synucleins: “Here, There and Everywhere”. Int. Rev. Cell Mol. Biol..

[15] Surguchev A.A., Surguchov A. (2017). Synucleins and gene expression: Ramblers in a crowd or cops regulating traffic?. Front. Mol. Neurosci..

[16] Iwai A., Masliah E., Yoshimoto M., Ge N., Flanagan L., de Silva H.A., Kittel A., Saitoh T. (1995). The precursor protein of non-a beta component of Alzheimer’s disease amyloid is a presynaptic protein of the central nervous system. Neuron.

[17] Soll L.G., Eisen J.N., Vargas K.J., Medeiros A.T., Hammar K.M., Morgan J.R. (2020). α-Synuclein-112 impairs synaptic vesicle recycling consistent with its enhanced membrane binding properties. Front. Cell Dev. Biol..

[18] Cremades N., Chen S.W., Dobson C.M. (2017). Structural characteristics of α-synuclein oligomers. Int. Rev. Cell Mol. Biol..

[19] Brás I.C., Xylaki M., Outeiro T.F. (2020). Mechanisms of alpha-synuclein toxicity: An update and outlook. Prog. Brain Res..

[20] Spillantini M.G., Schmidt M.L., Lee V.M., Trojanowski J.Q., Jakes R., Goedert M. (1997). Alpha-synuclein in lewy bodies. Nature.

[21] Papp M.I., Kahn J.E., Lantos P.L. (1989). Glial cytoplasmic inclusions in the CNS of patients with multiple system atrophy (Striatonigral degeneration, olivopontocerebellar atrophy and shy-drager syndrome). J. Neurol. Sci..

[22] Shahmoradian S.H., Lewis A.J., Genoud C., Hench J., Moors T.E., Navarro P.P., Castaño-Díez D., Schweighauser G., Graff-meyer A., Goldie K.N. (2019). Lewy pathology in Parkinson’s disease consists of crowded organelles and lipid membranes. Nat. Neurosci..

[23] Trinkaus V.A., Riera-Tur I., Martínez-Sánchez A., Bäuerlein F.J.B., Guo Q., Arzberger T., Baumeister W., Dudanova I., Hipp M.S., Hartl F.U. (2021). In situ architecture of neuronal α-synuclein inclusions. Nat. Commun..

[24] Mahul-Mellier A.-L., Burtscher J., Maharjan N., Weerens L., Croisier M., Kuttler F., Leleu M., Knott G.W., Lashuel H.A. (2020). The process of lewy body formation, rather than simply α-synuclein fibrillization, is one of the major drivers of neurodegeneration. Proc. Natl. Acad. Sci. USA.

[25] Cremades N., Cohen S.I.A., Deas E., Abramov A.Y., Chen A.Y., Orte A., Sandal M., Clarke R.W., Dunne P., Aprile F.A. (2012). Direct observation of the interconversion of normal and toxic forms of α-synuclein. Cell.

[26] Ghosh D., Singh P.K., Sahay S., Jha N.N., Jacob R.S., Sen S., Kumar A., Riek R., Maji S.K. (2015). Structure based aggregation studies reveal the presence of helix-rich intermediate during α-synuclein aggregation. Sci. Rep..

[27] Antonschmidt L., Dervişoğlu R., Sant V., Movellan K.T., Mey I., Riedel D., Steinem C., Becker S., Andreas L.B., Griesinger C. (2021). Insights into the molecular mechanism of amyloid filament formation: Segmental folding of α-synuclein on lipid membranes/molecular mechanism of αs filament folding on membranes. Sci. Adv..

[28] Iljina M., Garcia G.A., Horrocks M.H., Tosatto L., Choi M.L., Ganzinger K.A., Abramov A.Y., Gandhi S., Wood N.W., Cremades N. (2016). Kinetic model of the aggregation of alpha-synuclein provides insights into prion-like spreading. Proc. Natl. Acad. Sci. USA.

[29] Li X., Dong C., Hoff M., Garen C.R., Cort L.M., Petersen N.O., Woodside M.T., Hoffmann M., Garen C.R., Cortez L.M. (2019). Early stages of aggregation of engineered α-synuclein monomers and oligomers in solution. Sci. Rep..

[30] Buell A.K., Dobson C.M., Knowles T.P.J. (2014). The physical chemistry of the amyloid phenomenon: Thermodynamics and kinetics of filamentous protein aggregation. Essays Biochem..

[31] Gaspar R., Meisl G., Buell A.K., Young L., Kaminski C.F., Knowles T.P.J., Sparr E., Linse S. (2017). Secondary nucleation of monomers on fibril surface dominates α-synuclein aggregation and provides autocatalytic amyloid amplification. Q. Rev. Biophys..

[32] Buell A.K., Galvagnion C., Gaspar R., Sparr E., Vendruscolo M., Knowles T.P.J., Linse S., Dobson C.M. (2014). Solution conditions determine the relative importance of nucleation and growth processes in α-synuclein aggregation. Proc. Natl. Acad. Sci. USA.

[33] Meisl G., Knowles T.P.J., Klenerman D. (2020). The molecular processes underpinning prion-like spreading and seed amplification in protein aggregation. Curr. Opin. Neurobiol..

[34] Wells C., Brennan S., Keon M., Ooi L. (2021). The role of amyloid oligomers in neurodegenerative pathologies. Int. J. Biol. Macromol..

[35] Balchin D., Hayer-Hartl M., Hartl F.U. (2016). In vivo aspects of protein folding and quality control. Science.

[36] Kim Y.E., Hipp M.S., Bracher A., Hayer-Hartl M., Ulrich Hartl F. (2013). Molecular chaperone functions in protein folding and proteostasis. Annu. Rev. Biochem..

[37] Aguado A., Fernández-Higuero J.A., Moro F., Muga A. (2015). Chaperone-assisted protein aggregate reactivation: Different solutions for the same problem. Arch. Biochem. Biophys..

[38] Wentink A., Nussbaum-Krammer C., Bukau B. (2019). Modulation of amyloid states by molecular chaperones. Cold Spring Harb. Perspect. Biol..

[39] Nillegoda N.B., Wentink A.S., Bukau B. (2018). Protein disaggregation in multicellular organisms. Trends Biochem. Sci..

[40] Avellaneda M.J., Franke K.B., Sunderlikova V., Bukau B., Mogk A., Tans S.J. (2020). processive extrusion of polypeptide loops by a hsp100 disaggregase. Nature.

[41] Gates S.N., Gates S.N., Yokom A.L., Lin J., Jackrel M.E., Rizo A.N., Kendsersky N.M., Buell C.E., Sweeny E.A., Mack K.L. (2017). Ratchet-like polypeptide translocation mechanism of the AAA + disaggregase Hsp104. Science.

[42] Mogk A., Kummer E., Bukau B. (2015). Cooperation of Hsp70 and Hsp100 Chaperone Machines in Protein Disaggregation. Front. Mol. Biosci..

[43] Shorter J. (2011). The mammalian disaggregase machinery: Hsp110 synergizes with Hsp70 and Hsp40 to catalyze protein disaggregation and reactivation in a cell-free system. PLoS ONE.

[44] Desantis M.E., Leung E.H., Sweeny E.A., Jackrel M.E., Cushman-Nick M., Neuhaus-Follini A., Vashist S., Sochor M.A., Knight M.N., Shorter J. (2012). Operational plasticity enables Hsp104 to disaggregate diverse amyloid and nonamyloid clients. Cell.

[45] Lo Bianco C., Shorter J., Régulier E., Lashuel H., Iwatsubo T., Lindquist S., Aebischer P. (2008). Hsp104 antagonizes α-synuclein aggregation and reduces dopaminergic degeneration in a rat model of parkinson disease. J. Clin. Investig..

[46] Tariq A., Jackrel M.E., Lin J., Hesketh C.D., Carman P.J., Mack K.L., Weitzman R., Gambogi C., Murillo O.A.H., Sweeny E.A. (2019). Mining disaggregase sequence space to counter TDP-43, FUS, and a-synuclein proteotoxicity. Cell Rep..

[47] Rampelt H., Kirstein-Miles J., Nillegoda N.B., Chi K., Scholz S.R., Morimoto R.I., Bukau B. (2012). Metazoan Hsp70 machines use Hsp110 to power protein disaggregation. EMBO J..

[48] Nillegoda N.B., Kirstein J., Szlachcic A., Berynskyy M., Stank A., Stengel F., Arnsburg K., Gao X., Scior A., Aebersold R. (2015). Crucial HSP70 co-chaperone complex unlocks metazoan protein disaggregation. Nature.

[49] Kirstein J., Arnsburg K., Scior A., Szlachcic A., Guilbride D.L., Morimoto R.I., Bukau B., Nillegoda N.B. (2017). In vivo properties of the disaggregase function of j-proteins and Hsc70 in caenorhabditis elegans stress and aging. Aging Cell.

[50] Duennwald M.L., Echeverria A., Shorter J. (2012). Small heat shock proteins potentiate amyloid dissolution by protein disaggregases from yeast and humans. PLoS Biol..

[51] Gao X., Carroni M., Nussbaum-Krammer C., Mogk A., Nillegoda N.B., Szlachcic A., Guilbride D.L., Saibil H.R., Mayer M.P., Bukau B. (2015). Human Hsp70 Disaggregase Reverses Parkinson’s-Linked α-Synuclein Amyloid Fibrils. Mol. Cell.

[52] Kampinga H.H., Craig E.A. (2010). The HSP70 chaperone machinery: J proteins as drivers of functional specificity. Nat. Rev. Mol. Cell Biol..

[53] Cabrera Y., Dublang L., Fernández-Higuero J.A., Albesa-Jové D., Lucas M., Viguera A.R., Guerin M.E., Vilar J.M.G., Muga A., Moro F. (2019). Regulation of human Hsc70 ATPase and chaperone activities by Apg2: Role of the acidic subdomain. J. Mol. Biol..

[54] Zhuravleva A., Clerico E.M., Gierasch L.M. (2012). An interdomain energetic tug-of-war creates the allosterically active state in Hsp70 molecular chaperones. Cell.

[55] Kityk R., Vogel M., Schlecht R., Bukau B., Mayer M.P. (2015). Pathways of allosteric regulation in Hsp70 chaperones. Nat. Commun..

[56] Faust O., Abayev-Avraham M., Wentink A.S., Maurer M., Nillegoda N.B., London N., Bukau B., Rosenzweig R. (2020). Hsp40s employ class-specific regulation to drive Hsp70 functional diversity. Nature.

[57] Wentink A.S., Nillegoda N.B., Feufel J., Ubartaitė G., Schneider C.P., De Los Rios P., Hennig J., Barducci A., Bukau B. (2020). Molecular Dissection of Amyloid Disaggregation by Human HSP70. Nature.

[58] De Los Rios P., Ben-Zvi A., Slutsky O., Azem A., Goloubinoff P. (2006). Hsp70 chaperones accelerate protein translocation and the unfolding of stable protein aggregates by entropic pulling. Proc. Natl. Acad. Sci. USA.

[59] Sousa R., Liao H., Cuéllar J., Jin S., Valpuesta J.M., Jin A.J., Lafer E.M. (2016). Clathrin-coat disassembly illuminates the mechanisms of Hsp70 force generation. Nat. Struct. Mol. Biol..

[60] Bracher A., Verghese J. (2015). The nucleotide exchange factors of Hsp70 molecular chaperones. Front. Mol. Biosci..

[61] Franco A., Gracia P., Colom A., Camino J.D., Fernández-Higuero J.A., Orozco N., Dulebo A., Saiz L., Cremades N., Vilar J.M. (2021). All-or-none amyloid disassembly via chaperone-triggered fibril unzipping favors clearance of α-synuclein toxic species. Proc. Natl. Acad. Sci. USA.

[62] Chen S.W., Drakulic S., Deas E., Ouberai M., Aprile F.A., Arranz R., Ness S., Roodveldt C., Guilliams T., De-Genst E.J. (2015). Structural characterization of toxic oligomers that are kinetically trapped during α-synuclein fibril formation. Proc. Natl. Acad. Sci. USA.

[63] Fusco G., Chen S.W., Williamson P.T.F., Cascella R., Perni M., Jarvis J.A., Cecchi C., Vendruscolo M., Chiti F., Cremades N. (2017). Structural basis of membrane disruption and cellular toxicity by alpha-synuclein oligomers. Science.

[64] Schneider M.M., Gautam S., Herling T.W., Andrzejewska E., Vendruscolo M., Bracher A., Dobson C.M., Hartl F.U. (2020). The Hsc70 disaggregation machinery removes monomer units directly from α-synuclein fibril ends. bioRxiv.

[65] Ferrari L., Geerts W.J.C., Van Wezel M., Kos R., Van Bezouwen L.S., Förster F.G., Stefan G.D. (2018). Human chaperones untangle fibrils of the Alzheimer protein Tau. bioRxiv.

[66] Nachman E., Wentink A., Madiona K., Bousset L., Katsinelos T., Kampinga H., Mcewan W.A., Jahn T.R., Melki R., Mogk A. (2020). Disassembly of tau fibrils by the human Hsp70 disaggregation machinery generates small seeding-competent species. J. Biol. Chem..

[67] Scior A., Buntru A., Arnsburg K., Ast A., Iburg M., Juenemann K., Pigazzini M.L., Mlody B., Puchkov D., Priller J. (2018). Complete suppression of htt fibrilization and disaggregation of htt fibrils by a trimeric chaperone complex. EMBO J..

[68] Goedert M., Spillantini M.G., Jakes R., Rutherford D., Crowther R.A. (1989). Multiple isoforms of human microtubule-associated protein tau: Sequences and localization in neurofibrillary tangles of Alzheimer’s disease. Neuron.

[69] Rossi M., Baiardi S., Parchi P. (2019). Understanding prion strains: Evidence from studies of the disease forms affecting humans. Viruses.

[70] Adamcik J., Mezzenga R. (2018). Amyloid polymorphism in the protein folding and aggregation energy landscape. Angew. Chem. Int. Ed. Engl..

[71] Tycko R. (2015). Amyloid polymorphism: Structural basis and neurobiological relevance. Neuron.

[72] Gracia P., Camino J.D., Volpicelli-Daley L., Cremades N. (2020). Multiplicity of α-synuclein aggregated species and their possible roles in disease. Int. J. Mol. Sci..

[73] Guerrero-Ferreira R., Kovacik L., Ni D., Stahlberg H. (2020). New insights on the structure of alpha-synuclein fibrils using cryo-electron microscopy. Curr. Opin. Neurobiol..

[74] Li B., Ge P., Murray K.A., Sheth P., Zhang M., Nair G., Sawaya M.R., Shin W.S., Boyer D.R., Ye S. (2018). Cryo-EM of full-length α-synuclein reveals fibril polymorphs with a common structural kernel. Nat. Commun..

[75] Guerrero-Ferreira R., Mi Taylor N., Arteni A.-A., Kumari P., Mona D., Ringler P., Britschgi M., Lauer M.E., Makky A., Verasdonck J. (2019). Two new polymorphic structures of human full-length alpha-synuclein fibrils solved by cryo-electron microscopy. eLife.

[76] Boyer D.R., Li B., Sun C., Fan W., Sawaya M.R., Jiang L., Eisenberg D.S. (2019). Structures of fibrils formed by α-synuclein hereditary disease mutant h50q reveal new polymorphs. Nat. Struct. Mol. Biol..

[77] Zhao K., Li Y., Liu Z., Long H., Zhao C., Luo F., Sun Y., Tao Y., Su X., Li D. (2020). Parkinson’s disease associated mutation e46k of α-synuclein triggers the formation of a distinct fibril structure. Nat. Commun..

[78] Boyer D.R., Li B., Sun C., Fan W., Zhou K., Hughes M.P., Sawaya M.R., Jiang L., Eisenberg D.S. (2020). The α-synuclein hereditary mutation e46k unlocks a more stable, pathogenic fibril structure. Proc. Natl. Acad. Sci. USA.

[79] Sun Y., Hou S., Zhao K., Long H., Liu Z., Gao J., Zhang Y., Su X.-D., Li D., Liu C. (2020). Cryo-EM structure of full-length α-synuclein amyloid fibril with parkinson’s disease familial a53t mutation. Cell Res..

[80] Arakhamia T., Lee C.E., Carlomagno Y., Seyfried N.T., Petrucelli L., Fitzpatrick A.W.P., Arakhamia T., Lee C.E., Carlomagno Y., Duong D.M. (2020). Post-translational modifications mediate the structural diversity of tauopathy strains. Cell.

[81] Anderson J.P., Walker D.E., Goldstein J.M., De Laat R., Banducci K., Caccavello R.J., Barbour R., Huang J., Kling K., Lee M. (2006). Phosphorylation of ser-129 is the dominant pathological modification of alpha-synuclein in familial and sporadic lewy. J. Biol. Chem..

[82] Barrett P.J., Timothy Greenamyre J. (2015). Post-translational modification of α-synuclein in Parkinson’s disease. Brain Res..

[83] Sorrentino Z.A., Giasson B.I. (2020). The emerging role of α-synuclein truncation in aggregation and disease. J. Biol. Chem..

[84] Ni X., McGlinchey R.P., Jiang J., Lee J.C. (2019). Structural insights into α-synuclein fibril polymorphism: Effects of Parkinson’s disease-related C-terminal truncations. J. Mol. Biol..

[85] Li Y., Zhao C., Luo F., Liu Z., Gui X., Luo Z., Zhang X., Li D., Liu C., Li X. (2018). Amyloid fibril structure of α-synuclein determined by cryo-electron microscopy. Cell Res..

[86] González N., Arcos-López T., König A., Quintanar L., Menacho Márquez M., Outeiro T.F., Fernández C.O. (2019). Effects of alpha-synuclein post-translational modifications on metal binding. J. Neurochem..

[87] Zhao K., Lim Y.-J., Liu Z., Long H., Sun Y., Hu J.-J. (2020). Parkinson’s disease-related phosphorylation at tyr39 rearranges α-synuclein amyloid fibril structure revealed by cryo-EM. Proc. Natl. Acad. Sci. USA.

[88] Schweighauser M., Shi Y., Tarutani A., Kametani F., Murzin A.G., Ghetti B., Matsubara T., Tomita T., Ando T., Hasegawa K. (2020). Structures of alpha-synuclein filaments from multiple system atrophy. Nature.

[89] Fitzpatrick A.W.P., Falcon B., He S., Murzin A.G., Murshudov G., Garringer H.J., Crowther R.A., Ghetti B., Goedert M., Scheres S.H.W. (2017). Cryo-EM structures of tau filaments from Alzheimer’s disease. Nature.

[90] Falcon B., Zhang W., Murzin A.G., Murshudov G., Garringer H.J.G., Vidal R., Crowther R.A., Ghetti B., Scheres S.H.W., Goedert M. (2018). Structures of filaments from pick’s disease reveal a novel tau protein fold. Nature.

[91] Falcon B., Zivanov J., Zhang W., Murzin A.G., Garringer H.J., Vidal R., Crowther R.A., Newell K.L., Ghetti B., Goedert M. (2019). Novel tau filament fold in chronic traumatic encephalopathy encloses hydrophobic molecules. Nature.

[92] Zhang W., Tarutani A., Newell K.L., Murzin A.G., Matsubara T., Falcon B., Vidal R., Garringer H.J., Shi Y., Ikeuchi T. (2020). Novel tau filament fold in corticobasal degeneration, a four-repeat tauopathy. Nature.

[93] Fitzpatrick A.W.P., Saibil H.R. (2019). Cryo-EM of amyloid fibrils and cellular aggregates. Curr. Opin. Struct. Biol..

[94] Verma M., Vats A., Taneja V. (2015). Toxic species in amyloid disorders: Oligomers or mature fibrils. Ann. Indian Acad. Neurol..

[95] Olzscha H., Schermann S.M., Woerner A.C., Pinkert S., Hecht M.H., Tartaglia G.G., Vendruscolo M., Hayer-Hartl M., Hartl F.U., Vabulas R.M. (2011). Amyloid-like aggregates sequester numerous metastable proteins with essential cellular functions. Cell.

[96] Park S.H., Kukushkin Y., Gupta R., Chen T., Konagai A., Hipp M.S., Hayer-Hartl M., Hartl F.U. (2013). PolyQ Proteins interfere with nuclear degradation of cytosolic proteins by sequestering the sis1p chaperone. Cell.

[97] Hipp M.S., Park S.H., Hartl U.U. (2014). Proteostasis impairment in protein-misfolding and -aggregation diseases. Trends Cell Biol..

[98] Yu A., Shibata Y., Shah B., Calamini B., Lo D.C., Morimoto R.I. (2014). Protein aggregation can inhibit clathrin-mediated endocytosis by chaperone competition. Proc. Natl. Acad. Sci. USA.

[99] Labbadia J., Morimoto R.I. (2015). The biology of proteostasis in aging and disease. Annu. Rev. Biochem..

[100] Choe Y.J., Park S.H., Hassemer T., Körner R., Vincenz-Donnelly L., Hayer-Hartl M., Hartl F.U. (2016). Failure of RQC machinery causes protein aggregation and proteotoxic stress. Nature.

[101] Guo Q., Lehmer C., Martínez-Sánchez A., Rudack T., Beck F., Hartmann H., Pérez-Berlanga M., Frottin F., Hipp M.S., Hartl F.U. (2018). In situ structure of neuronal C9orf72 Poly-GA aggregates reveals proteasome recruitment. Cell.

[102] Baldwin A.J., Knowles T.P.J., Tartaglia G.G., Fitzpatrick A.W., Devlin G.L., Shammas S.L., Waudby C.A., Mossuto M.F., Meehan S., Gras S.L. (2011). Metastability of native proteins and the phenomenon of amyloid formation. J. Am. Chem. Soc..

[103] Carulla N., Caddy G.L., Hall D.R., Gairı M., Feliz M., Giralt E., Robinson C.V., Dobson C.M. (2005). Molecular recycling within amyloid fibrils. Nature.

[104] Knowles T.P.J., Waudby C.A., Devlin G.L., Cohen S.I.A., Aguzzi A., Vendruscolo M., Terentjev E.M., Welland M.E., Dobson C.M. (2009). An analytical solution to the kinetics of breakable filament assembly. Science.

[105] Cohen S.I.A., Linse S., Luheshi L.M., Hellstrand E., White D.A., Rajah L., Otzen D.E., Vendruscolo M., Dobson C.M., Knowles T.P.J. (2013). Proliferation of amyloid-β42 aggregates occurs through a secondary nucleation mechanism. Proc. Natl. Acad. Sci. USA.

[106] Meisl G., Yang X., Hellstrand E., Frohm B., Kirkegaard J.B., Cohen S.I.A., Dobson C.M., Linse S., Knowles T.P.J. (2014). Differences in nucleation behavior underlie the contrasting aggregation kinetics of the Aβ40 and Aβ42 peptides. Proc. Natl. Acad. Sci. USA.

[107] Scheidt T., Łapińska U., Kumita J.R., Whiten D.R., Klenerman D., Wilson M.R., Cohen S.I.A., Linse S., Vendruscolo M., Dobson C.M. (2019). Secondary nucleation and elongation occur at different sites on Alzheimer’s amyloid-β aggregates. Sci. Adv..

[108] Tittelmeier J., Sandhof C.A., Ries H.M., Druffel-augustin S., Mogk A., Bukau B., Nussbaum-Krammer C. (2020). The HSP 110/HSP 70 disaggregation system generates spreading-competent toxic α-synuclein species. EMBO J..

[109] Winkler J., Tyedmers J., Bukau B., Mogk A. (2012). Hsp70 targets hsp100 chaperones to substrates for protein disaggregation and prion fragmentation. J. Cell Biol..

[110] Sweeny E.A., Shorter J. (2016). Mechanistic and structural insights into the prion-disaggregase activity of Hsp104. J. Mol. Biol..

[111] Davis J.K., Sindi S.S. (2016). A mathematical model of the dynamics of prion aggregates with chaperone-mediated fragmentation. J. Math. Biol..

[112] Chernoff Y.O., Lindquist S.L., Ono B., Inge-Vechtomov S.G., Liebman S.W. (1995). Role of the chaperone protein Hsp104 in propagation of the yeast prion-like factor [Psi+]. Science.

[113] Taguchi Y.V., Gorenberg E.L., Nagy M., Thrasher D., Fenton W.A., Volpicelli-Daley L., Horwich A.L., Chandra S.S. (2019). Hsp110 mitigates α-synuclein pathology in vivo. Proc. Natl. Acad. Sci. USA.

[114] Nagy M., Fenton W.A., Li D., Furtak K., Horwich A.L. (2016). Extended survival of misfolded G85R SOD1-linked ALS mice by transgenic expression of chaperone Hsp110. Proc. Natl. Acad. Sci. USA.

[115] Stepanenko O.V., Sulatsky M.I., Mikhailova E.V., Stepanenko O.V., Povarova O.I., Kuznetsova I.M., Turoverov K.K., Sulatskaya A.I. (2020). Alpha-b-crystallin effect on mature amyloid fibrils: Different degradation mechanisms and changes in cytotoxicity. Int. J. Mol. Sci..

[116] Binger K.J., Ecroyd H., Yang S., Carver J.A., Howlett G.J., Griffin M.D.W. (2013). Avoiding the oligomeric state: αB-crystallin inhibits fragmentation and induces dissociation of apolipoprotein C-II Amyloid fibrils. FASEB J..

[117] Kandasamy G., Andréasson C. (2018). Hsp70–Hsp110 chaperones deliver ubiquitin-dependent and -independent substrates to the 26s proteasome for proteolysis in yeast. J. Cell Sci..

[118] Ye Y., Klenerman D., Finley D. (2019). N-Terminal ubiquitination of amyloidogenic proteins triggers removal of their oligomers by the proteasome holoenzyme. J. Mol. Biol..

[119] Cliffe R., Sang J.C., Kundel F., Finley D., Klenerman D., Ye Y. (2019). Filamentous aggregates are fragmented by the proteasome holoenzyme. Cell Rep..

[120] Sang J.C., Hidari E., Klenerman D., Meisl G., Ranasinghe R.T., Spillantini M.G. (2021). Super-resolution imaging reveals α-synuclein seeded aggregation in sh-sy5y cells. Commun. Biol..

[121] Marrero-Winkens C., Sankaran C., Schätzl H.M. (2020). from seeds to fibrils and back: Fragmentation as an overlooked step in the propagation of prions and prion-like proteins. Biomolecules.

[122] Xu Y., Propson N.E., Du S., Xiong W., Zheng H. (2021). Autophagy deficiency modulates microglial lipid homeostasis and aggravates tau pathology and spreading. Proc. Natl. Acad. Sci. USA.

[123] Sabath N., Levy-Adam F., Younis A., Rozales K., Meller A., Hadar S., Soueid-Baumgarten S., Shalgi R. (2020). Cellular proteostasis decline in human senescence. Proc. Natl. Acad. Sci. USA.

[124] Brehme M., Voisine C., Rolland T., Wachi S., Soper J.H., Zhu Y., Orton K., Villella A., Garza D., Vidal M. (2014). A chaperome subnetwork safeguards proteostasis in aging and neurodegenerative disease. Cell Rep..

[125] Mckinnon C., Tabrizi S.J. (2014). The ubiquitin-proteasome system in neurodegeneration. Antioxid. Redox Signal..

[126] Martinez-Lopez N., Athonvarangkul D., Singh R. (2015). Autophagy and aging. Adv. Exp. Med. Biol..

[127] Caldwell K.A., Caldwell G.A., Shorter J. (2014). Potentiated Hsp104 variants antagonize diverse proteotoxic misfolding events. Cell.

[128] Fare C.M., Shorter J. (2021). (Dis)solving the problem of aberrant protein states. Dis. Model Mech..

[129] Perni M., Flagmeier P., Limbocker R., Cascella R., Aprile F.A., Galvagnion C., Heller G.T., Meisl G., Chen S.W., Kumita J.R. (2018). Multistep inhibition of α-synuclein aggregation and toxicity in vitro and in vivo by trodusquemine. ACS Chem. Biol..

[130] Limbocker R., Mannini B., Ruggeri F.S., Cascella R., Xu C.K., Perni M., Chia S., Chen S.W., Habchi J., Bigi A. (2020). Trodusquemine displaces protein misfolded oligomers from cell membranes and abrogates their cytotoxicity through a generic mechanism. Commun. Biol..

[131] Santos J., Gracia P., Navarro S., Peña-Díaz S., Pujols J., Cremades N., Pallarès I., Ventura S. (2021). α-Helical peptidic scaffolds to target α-synuclein toxic species with nanomolar affinity. Nat. Commun..

